# Anti-PD1 therapies induce an early expansion of Ki67^+^CD8^+^ T cells in metastatic non-oncogene addicted NSCLC patients

**DOI:** 10.3389/fimmu.2024.1483182

**Published:** 2024-12-18

**Authors:** Alain Gelibter, Lucrezia Tuosto, Angela Asquino, Marco Siringo, Arianna Sabatini, Ilaria Grazia Zizzari, Angelica Pace, Fabio Scirocchi, Flavio Valentino, Serena Bianchini, Salvatore Caponnetto, Donatella Paoli, Filippo Bellati, Daniele Santini, Marianna Nuti, Aurelia Rughetti, Chiara Napoletano

**Affiliations:** ^1^ Division of Oncology, Department of Radiological, Oncological and Pathological Science, Policlinico Umberto I, “Sapienza” University of Rome, Rome, Italy; ^2^ Laboratory of Tumor Immunology and Cell Therapies, Department of Experimental Medicine, Sapienza University of Rome, Rome, Italy; ^3^ Department of Onco-hematology, Gene and Cell Therapy, Bambino Gesù Children’s Hospital-Istituti di Ricovero e Cura a Carattere Scientifico (IRCCS), Rome, Italy; ^4^ Laboratory of Seminology-Sperm Bank “Loredana Gandini”, Department of Experimental Medicine, “Sapienza” University of Rome, Rome, Italy; ^5^ Department of Medical and Surgical Sciences and Translational Medicine, Sant’Andrea University Hospital, Sapienza University of Rome, Rome, Italy

**Keywords:** NSCLC, immune checkpoint inhibitors, anti-PD-1, lymphocytes, CD137

## Abstract

Pembrolizumab (an anti-PD1 antibody) alone or combined with chemotherapy represented the standard of care for advanced non-oncogene addicted non-small cell lung cancer (NSCLC) patients. These therapies induced early modifications of the immune response impacting the clinical outcome. Identifying early changes in the immune system was critical to directing the therapeutic choice and improving the clinical outcome. In this study, we aim to analyze the activating and inhibiting immune cells of NSCLC patients before and during therapy to identify patients who will benefit from immunotherapies. Forty-eight NSCLC patients were analyzed before (T0) and after the first cycle of immunotherapy (T1), evaluating several activating (CD137^+^and PD1^+^), proliferating (Ki67^+^) and immunosuppressing immune subsets (Tregs: total, active, resting, and non-suppressive; MDSCs: PMN(Lox1^+^)-MDSC and M-MDSCs) by cytofluorimetry. Concurrently, 14 soluble immune checkpoints were analyzed by Luminex assay. Immunotherapy significantly increased the levels of Ki67^+^(total and CD8^+^) T cells, PMN(Lox1^+^)-MDSCs, non-suppressive Tregs (nsTregs), and soluble PD1 from T0 to T1 in the entire NSCLC population, while decreased active Tregs. These changes were partially attributed to responding patients who showed an increase of Ki67^+^ and CD8^+^T cells and nsTregs at T1. CD137^+^(total, CD8^+^, and CD4^+^) T cells and soluble LAG3 were predictor factors at T0 and T1. A low ratio of Tregs/CD137+ T cells and high levels of Ki67^+^CD137^+^ T cells positively correlated with response to therapy at T0 and T1, respectively. Results highlighted that immunotherapy improved the immunological fitness of those patients who benefited from immunotherapy, changing the immunological balance towards immune activation.

## 1 Introduction

Lung cancer has the second highest incidence and mortality cancer-related, with 20 million new cases and 79 million deaths per year. Non-small cell lung cancer (NSCLC) is the most common subtype, about 85% of all lung cancer types, and 40-50% of the patients are diagnosed with metastatic disease (1).

The introduction of immune checkpoint inhibitors (ICIs) has changed the treatment landscape of this type of cancer, improving patients’ survival (2, 3). In the metastatic non-oncogene addicted NSCLC group, the PD1/PD-L1 axis blockade has been introduced as monotherapy or in combination with chemotherapy in naïve patients according to the tumor expression of PD-L1 defined as Tumor Proportion Score (TPS). Indeed, the anti-PD1 antibody pembrolizumab is administered in patients with PD-L1≥50% (4). On the contrary, chemotherapy plus pembrolizumab (5, 6) or chemotherapy plus nivolumab and ipilimumab (an anti-CTLA4 antibody) represent the standard of care for patients with absent or low PD-L1 expression (TPS<50%) (7).

It is well known that immunotherapy early affects the immune system (8, 9). Peripheral blood immune cells were subjected to dynamic modifications induced by ICIs thus influencing the response to therapy and clinical outcome. In advanced NSCLC patients, early modifications in neutrophil-lymphocyte ratio (NLR) were predictors of response to ICIs. The decrease in NLR was associated with the activation of the IFNγ signature that drove the over-expression of genes related to antigen presentation and pro-inflammatory cytokines (10). In the same setting, anti-PD(L)-1 treatments were linked to an increase of effector memory T cells with novel TCRs and NK cells, contributing to a favorable clinical outcome and longer progression-free survival (PFS), respectively (11, 12).

In the last year, lymphocytes expressing CD137 have acquired great relevance in the anti-tumor immune response. CD137 (4-1BB) molecule is a TNFR family member, up-regulated by active T cells (13). This factor triggers T cell division and survival, promotes the functionality of T cells, the methylation of CD8 main genes, and protects against apoptosis (14, 15). CD137^+^ T cells were considered tumor-specific, and recently, we demonstrated that this cellular subset evaluated before therapy was an independent prognostic factor in advanced NSCLC patients who underwent immunotherapy as the first line (16). Together with the CD137 molecule, PD1 is another activating molecule of lymphocytes that identifies effector T cells. Eighty percent of the patients with increasing levels of PD1^+^CD8^+^ T cells after four weeks of anti-PD1 treatment exhibited clinical benefit and increased survival (17). CD137^+^ and PD1^+^ T cells combined with the Ki67 marker identified the proliferating activated lymphocyte subsets (18).

Also, the suppressive immune cells including regulatory T cells (Tregs) and myeloid-derived suppressive cells (MDSCs), both polymorphonuclear (PMN) Lox1^+^-MDSCs and monocytes (M)-MDSCs, were explored in NSCLC patients. The modification of Tregs during ICIs appeared to be controversial. Several authors demonstrated that Tregs (CD4^+^FOXP3^+^) were not modified after nivolumab treatment (19), while others showed a decrease in Tregs after 7 days of ICIs in patients who experienced pseudoprogression (20). MDSCs in the NSCLC setting were mostly studied as a prognostic factor and data regarding their modification after immunotherapy were scarce (21).

In addition to the cellular changes, several soluble factors, such as checkpoint molecules, were modified in blood by ICIs. In the metastatic NSCLC setting, the modulation of the soluble PD1 (sPD1) and PD-L1 (sPD-L1) was the most studied. Several authors demonstrated the association between sPD-L1 and a poor prognosis in patients treated with ICIs as second-line treatment (22, 23). We showed that responding patients to nivolumab treatment decreased the levels of sPD1 and sPD-L2, while non-responders showed an increase in the soluble lymphocyte activation genes (sLAG3) (24).

All these data underlined the ability of immunotherapy to affect several components of the immune system (cellular and soluble). Changes in each immunological parameter impact the immune balance (activation vs. suppression) of cancer patients and contribute to defining the clinical outcome of cancer patients.

In this study, we analyzed the changes in soluble immune checkpoints and different circulating T cell subsets belonging to the activatory (PD1^+^ and CD137^+^), proliferating (Ki67^+^) and inhibitory (Tregs, PMN(Lox1^+^)- and M-MDSCs) immune subsets in advanced non-oncogene addicted NSCLC patients before and after the first cycle of ICIs to highlight the relevance of continuously monitoring the immune fitness of cancer patients for the optimization of the therapeutic choice.

## 2 Materials and methods

### 2.1 Patients’ characteristics and inclusion criteria

Forty-eight patients affected by metastatic non-oncogene addicted non-small cell lung cancer (NSCLC) were enrolled between 2017 and 2023, at the Policlinico Umberto I. These patients were treated with ICIs according to Italian guidelines and AIFA approval. Patients with TPS≥50 received ICIs as monotherapy while those with TPS<50% received a combination of chemotherapy and ICIs, based on physicians’ choices. Criteria of inclusion: histologically diagnosis of non-oncogene addicted NSCLC; age > 18 years; Eastern Cooperative Oncology Group Performance Status (PS) scored between 0–2; adequate pulmonary, renal, liver, cardiac, and bone marrow function and symptomatic and stable central nervous system metastases. Criteria of exclusion: systemic immunosuppression, symptomatic interstitial lung disease and any significant comorbidity, autoimmune disease.

The disease control rate (DCR) was evaluated in each patient and was used to classify patients as responders (R) or non-responder patients (NR). R showed a complete, partial response or stable disease according to iRECIST criteria, while NR showed a progression as the best response.

The study was conducted following good clinical practice guidelines and the declaration of Helsinki and approved by the Ethics Committee of Policlinico Umberto I (Ethical Committee Protocol, RIF.CE: 4181).

### 2.2 Peripheral blood mononuclear cells and serum collection

Peripheral blood mononuclear cells (PBMCs) derived from 48 NSCLC patients treated with immunotherapy as first-line were collected before the therapy (T0) and after 3 weeks of treatment (T1). The cells were isolated from blood samples by Ficoll-Hypaque gradient (Lympholite-H, Ontario, Canada) by centrifugation at 1.800 rpm for 30 minutes. Concurrently, patients’ sera were collected using the BD Vacutainer Plus Plastic Serum tubes (Becton Dickinson, Franklin Lakes, New Jersey, U.S.) by centrifugation at 1.800 rpm for 10 minutes. PBMCs and sera were cryopreserved until use.

### 2.3 Immunophenotyping

PBMCs were characterized by multi-parametric flow cytometry combining different conjugated anti-human monoclonal antibodies (MoAbs) to evaluate distinct cell subsets. The MoAbs anti-CD3-BV510 (HIT3a clone), CD8-APC-H7 (SK1 clone), CD137 (4-1BB)-APC (4B4-1 clone), PD1-BB700 (EH12.1 clone), CD45RA-BB515 (HI100 clone), Ki67-BV421 (B56 clone) (all from BD Biosciences, San Jose, CA), and CCR7-PE (G043H7 clone) (BioLegend, San Diego, CA) were employed for the analysis T cells. Tregs were evaluated by anti-CD3 BV510 (HIT3A clone), anti-CD4-APC-H7 (RPA-T4 clone), CD45RA-BB515 (HI100 clone) (all from BD Biosciences), CD25-PE (MA251clone) (BioLegend), FOXP3-APC (PCH101 clone) (Thermo Fisher Scientific, Waltham, MA, USA) MoAbs. To study MDSCs the following MoAbs were used: anti-CD66b-PeCy7 (DREG-5 clone, BD Biosciences), anti-HLA-DR-FITCH (L243 clone), anti-CD45-AF700 (2D1 clone), anti-CD11c-BV421 (3.9 clone), anti-LOX1-PE (15C4 clone), anti-CD14-BB700 (MφP9 clone), anti-CD15-APC (HI98 clone) (all from Biolegend). LIVE/DEAD Fixable Yellow Dead Cell Staining kit (Thermo Fisher) was employed to identify live cells, and the Foxp3/Transcription Factor Staining Buffer Set (Invitrogen, Waltham, MA), to evaluate the intracellular staining (both for Ki67 and FoxP3). In analyzing active and proliferating T cells (CD137^+^, PD1^+^, and Ki67^+^ cells), lymphocytes were selected by gating on FSC-A/SSC-A, and SSC-A/CD3 and live cells/CD3^+^. Ten thousand live cells/CD3^+^ events were acquired and analyzed. Tregs were evaluated by gating on FSC-A/SSC-A, SSC-A/CD3^+^, live cells/CD3^+^, and SSC-A/CD4^+^ cells. Twenty thousand SSC-A/CD4^+^ cells were acquired. Regulatory T cells were identified as CD4^+^CD25^+^FoxP3^+^cells. Treg subpopulations were studied using the CD45RA marker as active (CD4^+^CD25^high^Foxp3^high^CD45RA^-^), resting (CD4^+^CD25^+^Foxp3^low^CD45RA^+^), and non-suppressive (CD4^+^CD25^low^Foxp3^low^CD45RA^-^) Tregs. MDSCs were selected by gating all events, except debris, using FSC-A/SSC-A parameters, followed by live cells/FSC-A. PMN(Lox1^+^)MDSCs were then selected as SSC-A/CD66b^+^ cells, HLA-DR^-^/CD66b ^+^ cells and CD66b^+^/Lox1^+^. M-MDSCs were recognized among CD66b^-^ cells, SSC-A/CD14^+^ and CD14^+^/HLA-DR^-^ cells. The negative controls were acquired by fluorescence minus one (FMO) and autofluorescence. All the samples were acquired by DxFLEX Flow Cytometer (Beckman Coulter, Brea, CA) and analyzed by FlowJo software (version 10.8.8, Becton Dickinson).

### 2.4 Measure of soluble cytokines and immune checkpoints

Soluble immune checkpoints were analyzed using the Immuno-Oncology Checkpoint 14-Plex Human ProcartaPlex Panel (ThermoFisher Scientific). The panel included 14 immune checkpoints i.e. BTLA, GITR, HVEM, IDO, LAG-3, PD1, PD-L1, PD-L2, TIM-3, CD28, CD80, CD137, CD27, CD152. All these analytes were evaluated by Luminex multiple assays and analyzed using Bioplex Manager MP software (Bio-Rad, Hercules, CA, USA).

### 2.5 Statistical analysis

Statistical analysis was performed using GraphPad Prism version 10 (GraphPad Software, Inc. San Diego, USA). Descriptive statistics (average and standard error media (SEM)) were used to describe the data groups. Student’s paired t-test was used to compare the two groups. Correlations among different cell subsets were assessed through Spearman’s rank correlation test. p<0.05 was considered significant.

For the sample size calculation, assuming an α-level of 0.05 and a β-level of 0.20 (power 80%), the required sample size was 44 cases to detect an increase of 35% in the Ki67^+^CD8^+^ lymphocyte subset from T0 to T1. The total number was increased to 48 to take into consideration patient loss at follow-up.

## 3 Results

### 3.1 Patients’ characteristics

Forty-eight patients with advanced non-oncogene addicted NSCLC were enrolled in this study as described in **Table 1**. Briefly, 19 patients were treated with ICIs alone (TPS≥50%) and twenty-nine in association with chemotherapy (TPS<50%). Pembrolizumab plus chemotherapy (4 cycles) was administered in 23% of patients, while 38% were treated with ipilimumab/nivolumab plus 2 cycles of chemotherapy. Twenty-four patients had PS=0 (50%), and 24 were scored as PS≥1 (PS=1: 19; PS=2: 5). Seven and 10 patients showed only liver or brain metastasis, respectively, while both liver and brain metastasis were observed in 4 patients. Twenty-seven patients had metastasis in other sites such as lymph nodes, lung, peritoneum, ovary, bone marrow, and soft tissues. Most patients were current or former smokers (83%), and 17% of patients declared never smoked. The disease control rate was used to classify responder (R) (67%) and non-responder (NR) (33%) patients after 3 months of the beginning of immunotherapy.

**Table 1:** Patients’ characteristics.

	n° (%)
Tot	48 (100)
Sex	
Male	38 (79)
Female	10 (21)
Age	
Median range	69,5
<75	35 (73)
≥75	13 (27)
Histotype	
Adenocarcinoma	38 (79)
Squamous	10 (21)
EOCG Performance Status	
0	24 (50)
≥1	24 (50)
Therapy for metastatic disease	
Pembrolizumab	19 (39)
Pembrolizumab+CHT	11 (23)
Ipi/Nivo+CHT	18 (38)
Metastasis	
Liver	7 (14)
Brain	10 (21)
Liver and Brain	4 (8)
other	27 (57)
Smoking status	
smoker (current/former)	40 (83)
non-smoker	8 (17)
Response to ICI	
yes	32 (67)
no	16 (33)

CHT, chemoterapy; Ipi, Ipilimumab; Nivo, Nivolumab; ICI, Immune checkpoint inhibitors.

### 3.2 Immunotherapy increased the levels of cytotoxic Ki67^+^T cells

**Figure 1:**
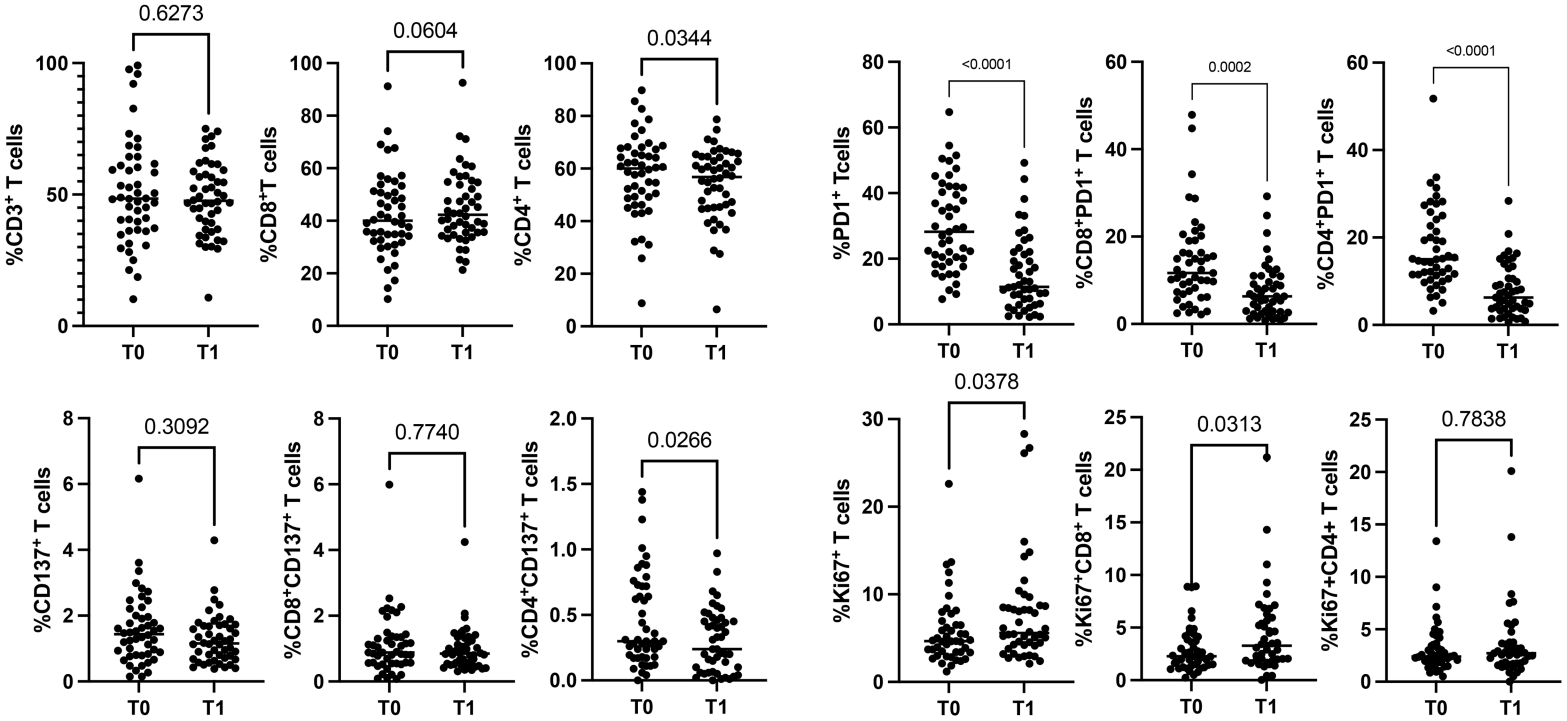
Modulation of CD3^+^, CD137^+^, PD1^+^, and Ki67^+^ T cell subsets during ICIs in 48 NSCLC patients evaluated by cytofluorimetry. The scattered dot plots display the values of CD3^+^ (total, CD8^+^, and CD4^+^) (top, left), CD137^+^ (total, CD8^+^, and CD4^+^) (lower, left), PD1^+^ (total, CD8^+^, and CD4^+^) (top, right) and Ki67^+^ (total, CD8^+^, and CD4^+^) (lower, right) T cells before (T0) and after the first cycle of therapy (T1). The horizontal lines correspond to the median values of the percentage of each T cell subset at T0 and T1. p values <0.05 were considered significant.

To evaluate the impact of immunotherapy on naïve NSCLC patients, we analyzed the circulating levels of CD3^+^, CD137^+^, PD1^+^, and Ki67^+^ T cells subsets (total, CD4^+^ and CD8^+^) of 48 patients before (T0) and after the first cycle of immunotherapy (T1) (**Figure 1**, **Supplementary Figure 1**). Results demonstrated that ICI treatments did not affect the levels of white blood cells (WBCs) and lymphocytes evaluated as absolute numbers and as a percentage of total CD3^+^ cells (n° WBCs/µL, T0 vs. T1: 9898±3879 vs. 9844±4190; n° lymphocytes/µL, T0 vs. T1: 1988±675 vs. 1798±630; %CD3^+^ cells: 51.2±20.29 vs. 48.6±13.8). However, the CD8^+^ and CD4^+^ T cell analysis, evaluated on the percentage of CD3^+^ cells, revealed that the CD4^+^ T cell subset was significantly reduced after therapy (p=0.03). In contrast, CD8^+^ T cells increased, even if this modulation was not statistically significant (p=0.06) (**Figure 1**). Similar changes were observed in the CD137^+^ T cell subset. Only CD4^+^CD137^+^ T cells significantly decreased after ICI treatment (p=0.02), while the entire population of CD137^+^ T cells and the CD8^+^CD137^+^ T cell subsets were not modified. Different results were obtained for PD1^+^ T cells (total, CD4^+^ and CD8^+^) that significantly decreased during treatment, suggesting that the presence of the therapeutic anti-PD1 antibodies (pembrolizumab or nivolumab) on the cells could block the ligation of the anti-PD1 antibody used for the analysis. Moreover, the evaluation of Ki67^+^ T cells demonstrated that ICIs increased the levels of proliferating T cells (Ki67^+^) (p=0.03), this increase was ascribed to the CD8^+^ T cell subset (p=0.03). At the same time, no modification was observed in CD4^+^ T cells. Finally, the percentage of naïve, effector, central memory, and effector memory was also analyzed, but no significant differences were obtained (data not shown).

### 3.3 CD8^+^ and Ki67^+^ T cells significantly increased after the first cycle of immunotherapy in responding patients

**Figure 2:**
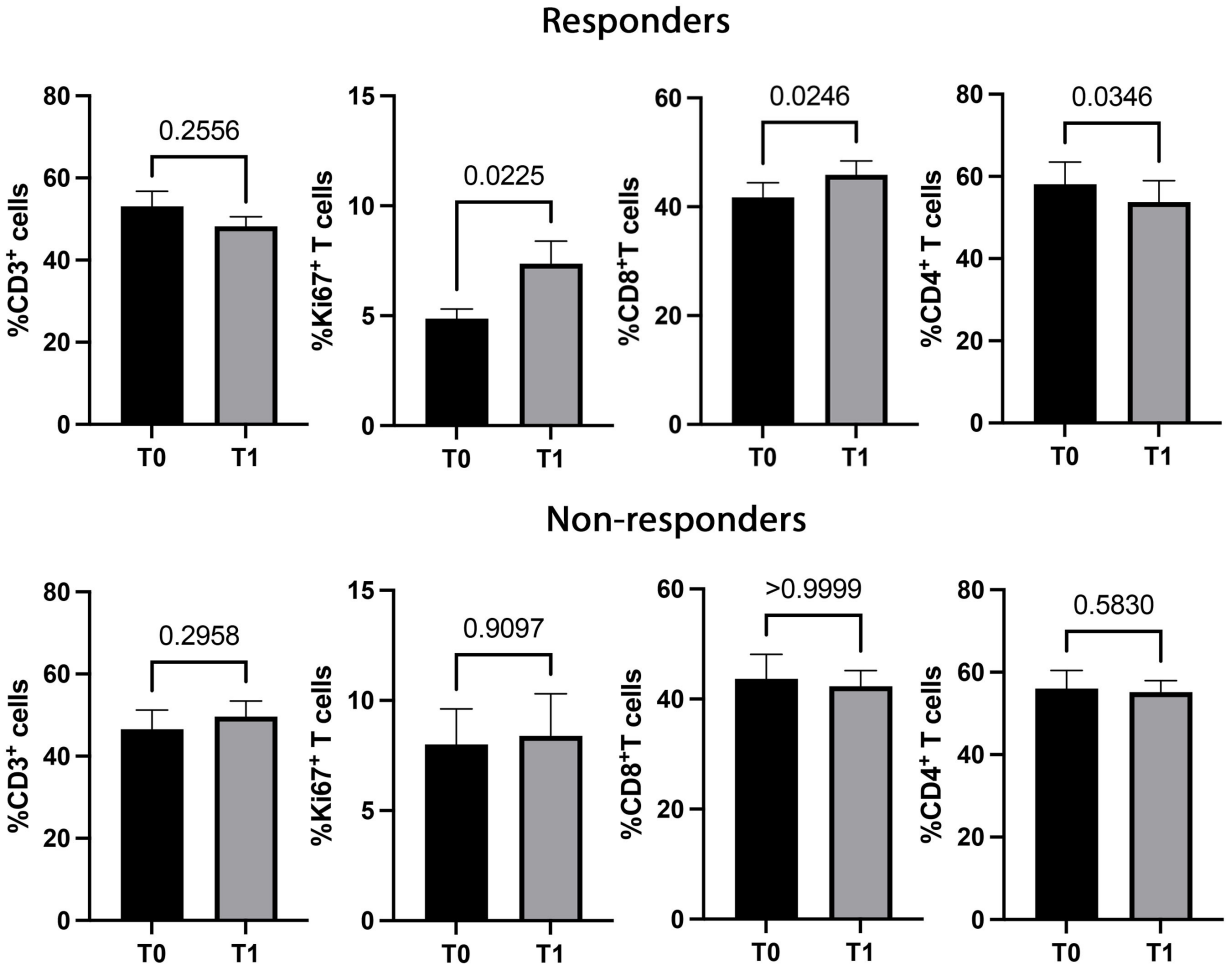
CD3^+^, Ki67^+^, CD8^+^, and CD4^+^ T cell subsets evaluated before and during therapy in responding and non-responding patients. The histograms represent the median values of the percentage CD3^+^, Ki67^+^, CD8^+^, and CD4^+^ T cell subsets in 32 responders (upper) and 16 non-responders (lower). The black histograms correspond to the values analyzed at baseline (T0) and the grey histograms correspond to the values analyzed after the first cycle of ICIs (T1) ± SEM. p values <0.05 were considered significant.

**Figure 3:**
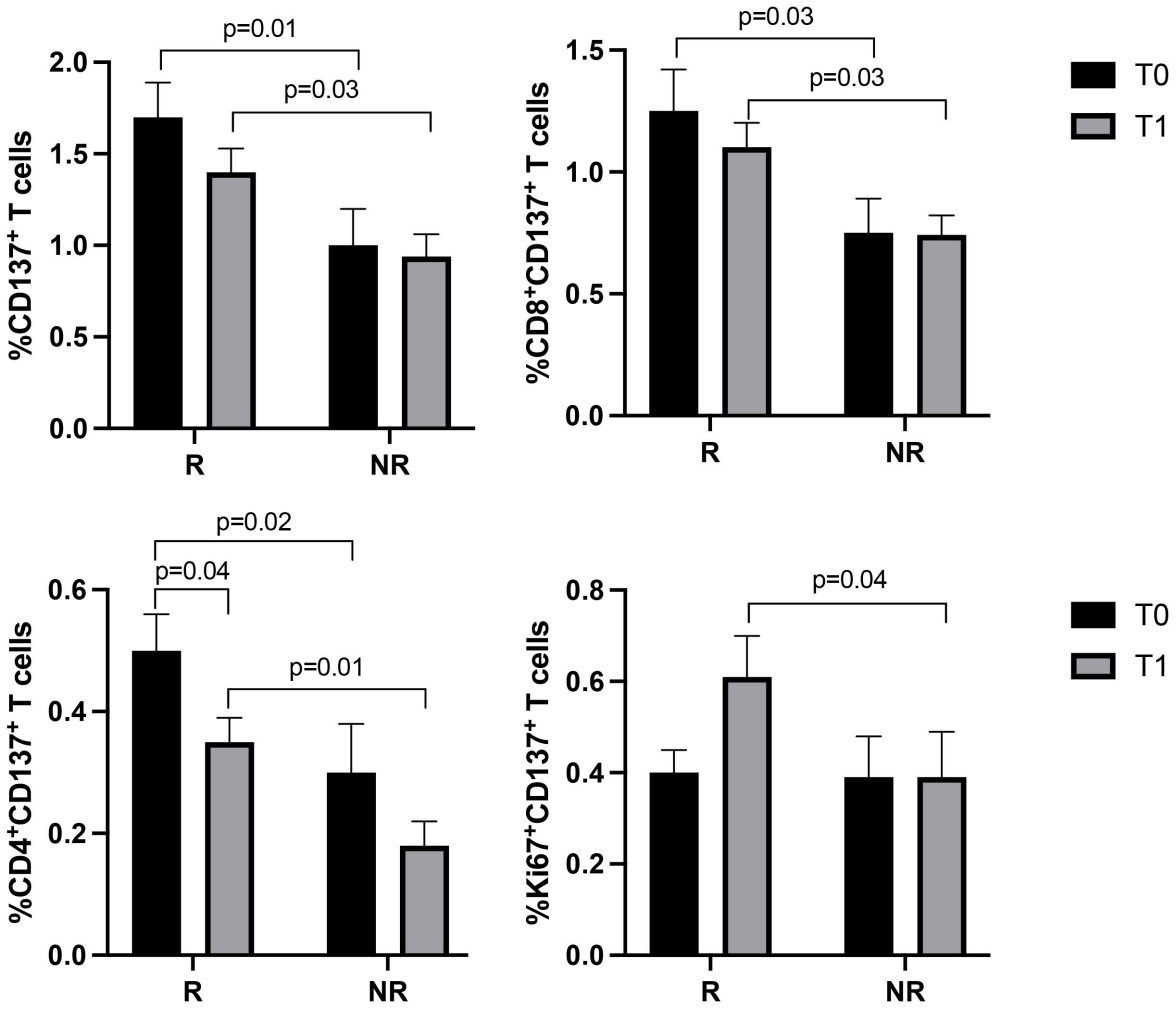
CD137^+^ (total, CD8^+^, CD4^+^, and Ki67^+^) T cell subsets evaluated by cytofluorimetry before and during therapy in responders and non-responders. The histograms represent the median values of the percentage CD137^+^ (total, CD8^+^, CD4^+^, and Ki67^+^) T cell subsets in responders (R) and non-responders (NR). The black histograms correspond to the values analyzed at baseline (T0) and the grey histograms correspond to the values analyzed after the first cycle of ICIs (T1) ± SEM. p values <0.05 were considered significant.

T cell subsets, such as CD3^+^ (CD8^+^ and CD4^+^), Ki67^+^, PD1^+^, and CD137^+^T cells, were further analyzed according to the response to therapy (**Figures 2**, **3**). Although the levels of CD3^+^ cells did not differ between responding and non-responding patients, the responding group showed a significant increase in Ki67^+^ (p=0.02) and CD8^+^ (p=0.02) T cells after the first treatment and a decrease in CD4^+^ T cells (p=0.03) (**Figure 2**). These modulations between T0 and T1 were not present in non-responding patients. Also, the PD1^+^ T cells significantly decreased at T1, although independently of the response (data not shown). Despite these changes observed in the responding group, responders and non-responders showed similar levels of Ki67^+^, CD8^+^ and CD4^+^ T cells before and after therapies (T0, R vs. NR: Ki67^+^ 5.4±3.3 vs 8±5.8, p=0.2; CD8^+^ 41.7±15.6 vs 43.7±16.7, p=0.7; CD4^+^ 58±15.6 vs 56±16.5, p=0.6; T1, R vs. NR: Ki67^+^ 7.2±5.6 vs 8.3±6.9, p=0.8; CD8^+^ 45.9±14.7 vs 42.3±10, p=0.6; CD4^+^ 53.7±15 vs 90±132, p=0.7). When these analyses were carried out in the CD137^+^ T cell subsets (**Figure 3**), results showed that responding patients had higher levels of CD137^+^ T cells (total, CD8^+^, and CD4^+^) compared to non-responders before and after the beginning of immunotherapy. On the contrary, no cellular changes were observed during therapy in the group of responders and non-responders except for CD137^+^ CD4^+^ T cells that decreased at T1 only in the responding group (p=0.04). Moreover, the analysis of Ki67^+^CD137^+^ T cells revealed that at T1, responding patients showed higher levels of proliferating CD137^+^ T cells than non-responders (p=0.04), despite both groups of patients having similar levels of Ki67^+^CD137^+^ T cells before the beginning of immunotherapy (T0 vs T1, R: 0.4±0.3 vs 0.6±0.5, p=0.1; NR: 0.39±0.39 vs 0.39±0.35, p=0.9) (**Figure 3**).

### 3.4 Active Tregs significantly decreased during immunotherapy

**Figure 4:**
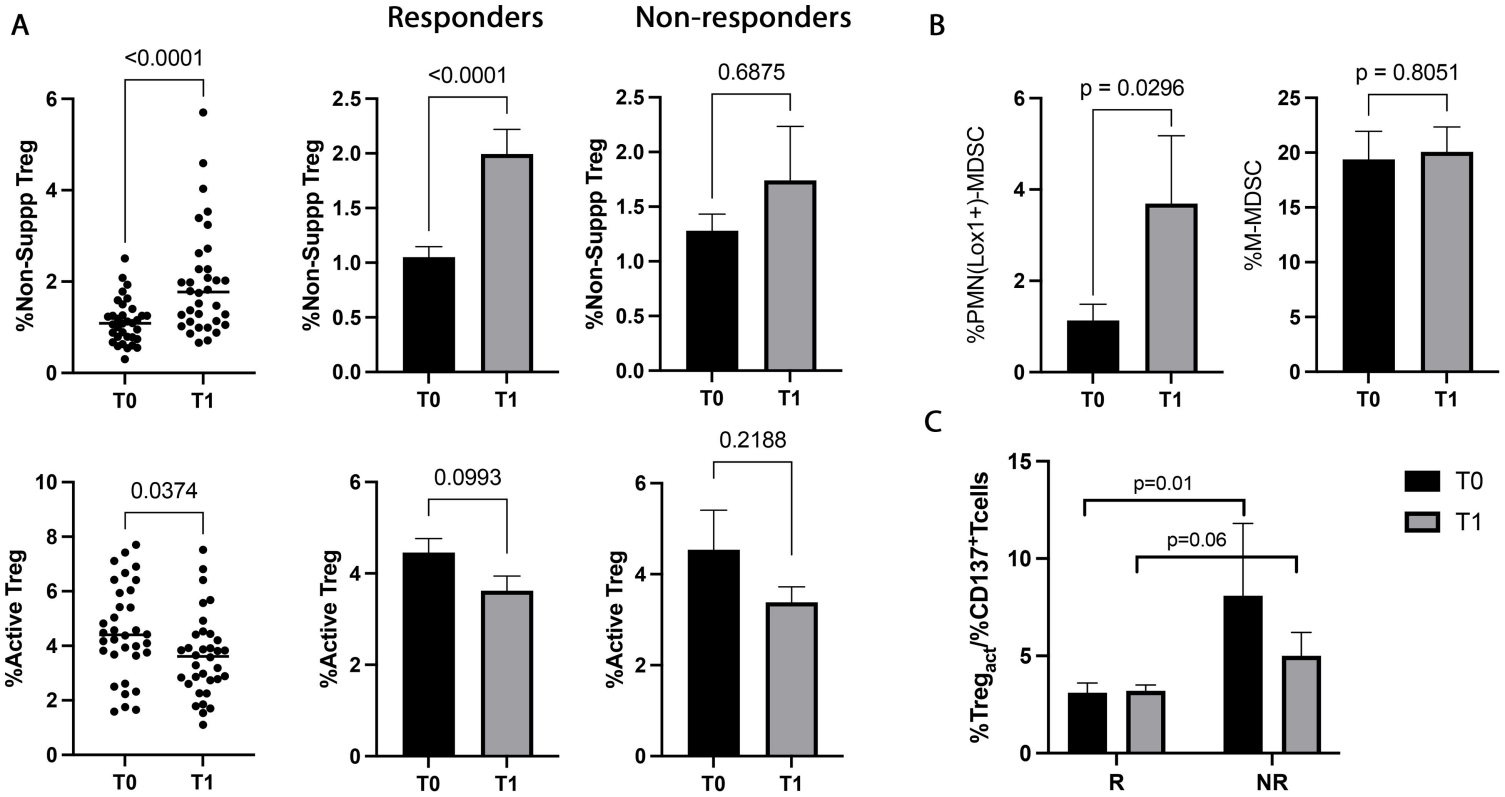
Treg and MDSC modulation evaluated in 48 NSCLC patients during immunotherapy. **(A)** The scattered dot plots display the values of the percentage of non-suppressive (upper), and active (lower) Tregs in NSCLC patients under ICIs before (T0) and after the first cycle of treatment (T1). The histograms showed the median levels of the percentage of non-suppressive and active Tregs at T0 (black histograms) and T1 (gray histograms) ± SEM. **(B)** Histograms showed the percentage of PMN(Lox1^+^)-MDSCs and M-MDSCs evaluated at T0 (black histograms) and T1 (gray histograms) ± SEM. **(C)** Histogram represented the %active Treg/%CD137^+^ T cells ratio in responders and non-responders. The black histograms display the median value of the ratio at T0, and the gray histogram is the ratio median value at T1± SEM. p values <0.05 were considered significant.

NSCLC patients were also analyzed for the immunosuppressive counterpart evaluating the percentage of circulating Tregs (total, active, resting, and non-suppressive) and MDSCs (PMN(Lox1^+^)- and M-MDSCs) before and during therapy (**Figure 4**). ICI treatment induced a significant increase in non-suppressive Tregs at T1 (p<0.0001). This change was ascribed to the responding group that showed a pronounced increase of non-suppressive Treg during treatment (p<0.0001). Moreover, a notable decrease was also observed in the active Treg subset at T1. This decrease was more prominent in the responders, even if it was not statistically significant (p=0.09) (**Figure 4A**). The analyses carried out in non-responding patients between T0 and T1 did not show changes in the non-suppressive and active Tregs. Similarly, total and resting Treg did not show any significant changes between T0 and T1 (T0 vs T1, totTreg: 7±2.2 vs 7.1±2.7, p=0.8; restTreg: 1.3±0.8 vs 1.4±0.7, p=0.2) and according to the response (T0, R vs NR: totTreg: 6.8±2 vs 7.2±2.5, p=0.8; restTreg: 1.2±0.8 vs 1.3±0.4, p=0.2; T1, totTreg: 7±3 vs 6.8±1.7, p>0.9; restTreg: 1.5±0.7 vs 1.5±0.7, p=0.9).

The data obtained from the evaluation of MDSCs (**Figure 4B**) revealed that the highly immunosuppressive PMN(Lox1^+^)-MDSC subset increased during therapy (p=0.02), although it did not correlate with the response. On the contrary, no significant modulation was found in the M-MDSC subset.

When the balance between immunosuppression/immune activation was analyzed as a percentage of active Treg or PMN(Lox1^+^)-MDSCs/CD137^+^ T cells ratio (**Figure 4C**), resulted that responding patient had at baseline a lower ratio of Tregs/CD137 compared to non-responders (p=0.01). This difference was also present after the first cycle of immunotherapy, even if it was not statistically significant (p=0.06). Treg/CD137^+^ T cells ratio evaluated at T0 and T1 did not show any significant differences in responders and non-responders (T0 vs T1, R: 3.1±2.8 vs 3.4±2, p>0.6; NR: 8.1±9.9 vs 5±3.2, p>0.5). Similar results were obtained by analyzing the PMN(Lox1^+^)-MDSCs/CD137^+^ T cells ratio (data not shown).

Moreover, the correlation between the cells involved in the immune activation and suppression showed that non-suppressive Treg positively correlated with Ki67^+^CD137^+^ T cells before and during therapy (T0: r=0.4, p=0.006; T1: r=0.4, p=0.003).

### 3.5 Soluble PD1 was modulated during ICI treatment

**Figure 5:**
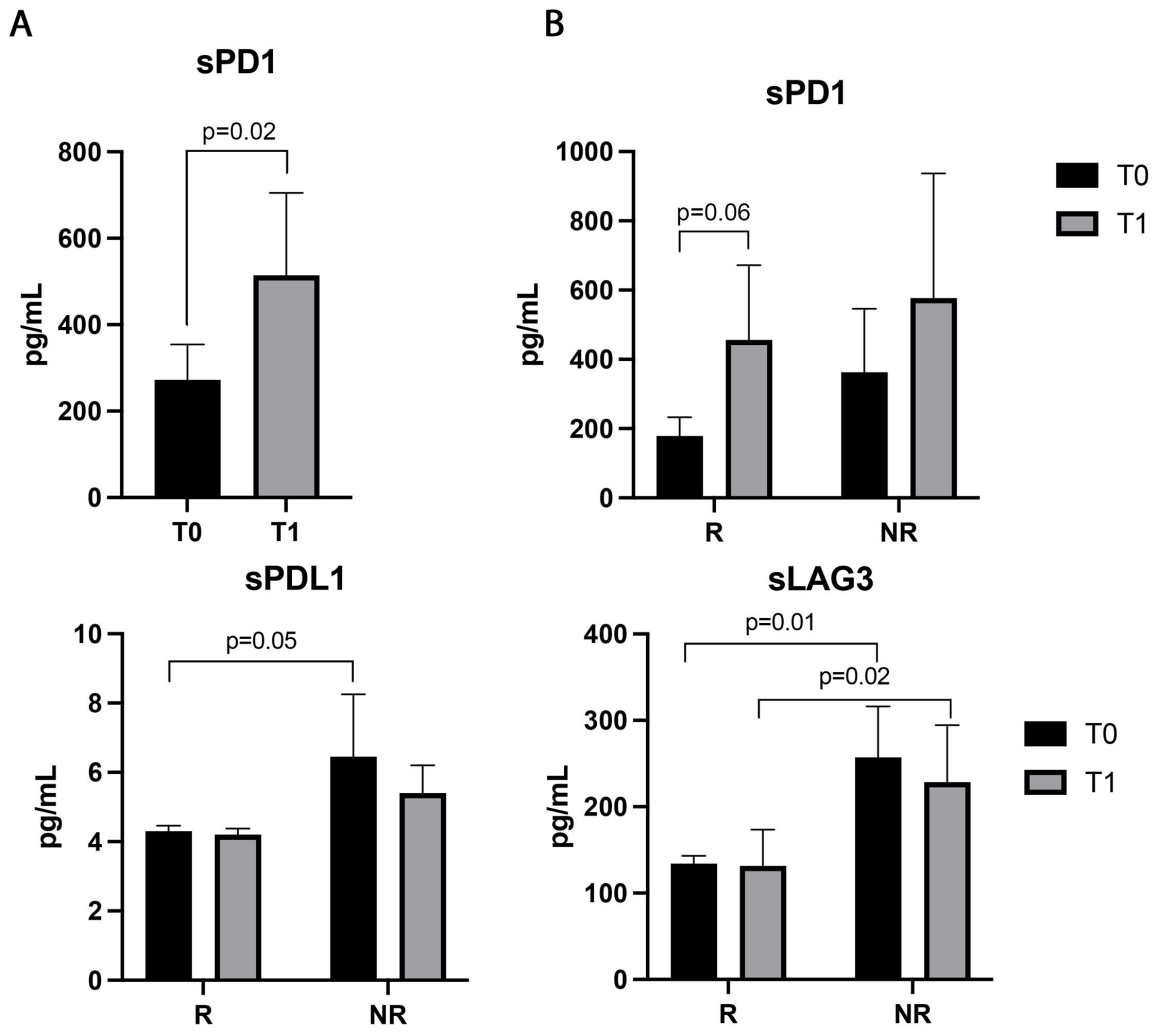
Soluble PD1, LAG3, and PDL1 evaluated in the sera of 48 NSCLC patients before and during immunotherapy. **(A)** Histograms represent the median values of the concentrations (pg/mL) of soluble PD1 before (T0, black histograms) and after the first cycle of ICIs (T1, gray histograms) ± SEM. **(B)** Histograms represent the median values of the concentrations (pg/mL) of soluble PD1, PDL1and LAG3 analyzed in responders (R) and non-responders (NR), before (T0, black histograms) and after the first cycle of immunotherapy (T1, gray histograms) ± SEM. B) p values <0.05 were considered significant.

Patients’ sera were also evaluated for 14 soluble immune checkpoints (**Figure 5**). The sPD1 was the only immune factor modulated during ICIs, significantly increasing after the first treatment cycle (p=0.02). Responding patients displayed an increasing trend of this parameter at T1, which was not statistically significant (p=0.06, **Figure 5B**). No significant differences in sPD1 levels between R and NR were found at baseline and T1 (**Figure 5B**). On the contrary, sPDL1 was not modulated by therapy, however, responders showed lower levels of sPDL1 at baseline than non-responders (p=0.05). Similar results were obtained with sLAG3 which was significantly lower in responder patients before (p=0.01) and during therapy (p=0.02) (**Figure 5B**). Both sPDL1 and LAG3 did not show differences between T0 and T1 in the groups of responders and non-responders (sPDL1: T0 vs T1, R: 4.2±0.8 vs 4.2±0.7, p=0.3; NR: 5.3±2.7 vs 4.6±0.7 p>0.6; sLAG3: T0 vs T1 R: 131.8±42.37 vs 134±41.49, p=0.6; NR: 228±210 vs 152±45 p>0.6).

## 4 Discussion

The clinical outcome of NSCLC patients treated with immunotherapy reflects the modification induced by ICIs on peripheral immune cells that impact the tumor microenvironment. The early identification of the immunotherapy effects on the immune system of cancer patients could be relevant for optimizing therapeutic strategies and identifying resistant patients.

In this study, we evaluated the changes in the immune system induced by ICIs in non-oncogene-addicted NSCLC patients undergoing immunotherapy as first-line treatment for identifying selected immune populations able to predict the response to anti-PD1 therapies. Results showed that these treatments rapidly expanded CD8^+^T cells (Ki67^+^CD8^+^ lymphocytes), suggesting that immunotherapy mainly acted on cytotoxic T cells enhancing their proliferation. In line with our results, similar effects of anti-PD(L)-1 therapies were frequently observed in NSCLC patients. Indeed, Kamphost et al. (17) demonstrated the presence of a high frequency of Ki67^+^PD1^+^CD8^+^ T cells after 6 cycles of immunotherapy. These lymphocytes were associated with an effector-like phenotype and a lower disease progression, suggesting the activation of tumor-specific CD8^+^ T cells. These proliferating cells, in addition to the PD1 molecule, expressed high levels of CTLA-4, and this phenomenon makes these cells particularly relevant in the anti-tumor immunity when the anti-PD1 therapies were combined with the anti-CTLA4 antibodies, as shown in melanoma patients (25, 26). Similar results were also observed in the neoadjuvant setting of NSCLC, in which responding patients enhanced the amount of Ki67^+^CD8^+^ T cells after chemoimmunotherapy (27), confirming that circulating cells could be considered an effective *reservoir* of T cells activated against the tumor. Moreover, proliferating CD8^+^ T cells with a high cytotoxic potential were also observed in stage IV NSCLC patients under anti-PD(L)-1 therapies. These cells showed the characteristics of exhausted T cells, which could be reinvigorated after immunotherapy (11).

Interestingly, we also observed that Ki67^+^ and CD8^+^ lymphocytes significantly increased during therapy only in the responding patients, although the overall amount of these populations was similar between responders and non-responders. These results confirm immunotherapy, only administered to patients with a less compromised immune system, could reinvigorate the pre-existing cytotoxic T cell subset relevant to anti-tumor activity. By comparing T cell subset levels of cancer patients at T0 with those of healthy donors (data not shown), we observed similar levels of T cell populations, further confirming this hypothesis. Moreover, when we evaluated the modulation of lymphocytes in the overall NSCLC population, we observed that the absolute number of these cells was homogeneous among patients and from T0 to T1, supporting the idea that each modification of immune cells obtained from our analyses was absolute, allowing us to compare the percentages of specific T cell subpopulations without misleading interpretation.

It is well known that the response of cancer patients to ICIs strongly depends on a delicate balance between immune activation and immune suppression. Among the immune-activating cell subsets, we analyzed the modulation of CD137^+^ T cells during ICI administration. This cell subset was described as the most functional tumor-specific and was associated with optimal performance status of immune fitness (28, 29), as demonstrated by high levels of these cells in healthy donors (29). Here, we observed for the first time that the CD137^+^ T cell populations (total, CD8^+^, and CD4^+^) remained at high levels in the responder group during therapy, maintaining their predictive role at baseline and after the first treatment cycle. Interestingly, after ICIs, responding patients showed high levels of proliferating CD137^+^ T cells, although responders and non-responders showed similar levels of this population at baseline. These results suggest that responders were equipped with an immunological tool that was more prone to reacting against tumors than non-responders. After the first cycle, the increasing levels of Ki67^+^CD137^+^ T cells further confirmed this hypothesis. The critical role of CD137^+^ T cells in the balance of immune response has been described in NSCLC and other solid tumors. Recently, we demonstrated the predictive and prognostic role of this population in advanced NSCLC patients under ICIs as first-line treatment (16). We showed that CD137^+^ T cells evaluated at baseline correlated with favorable survival in terms of PFS and OS and had a beneficial significance in terms of prognosis. In head and neck squamous cell carcinoma (HNSCCs) and metastatic renal cell carcinomas, the levels of CD137^+^ T cells were associated with response to therapy and prolonged survival (30, 31). In ovarian cancer, high tumor-infiltrating (TILs) CD137^+^ T cells correlated with improved survival (32). Moreover, several evidence regarding the anti-tumor potential of CD137^+^ T cells supported the introduction of the anti-CD137 agonistic antibodies in phase I and II clinical trials in monotherapy and combination with encouraging results (33), confirming the hypothesis that this cellular subset could be relevant for the tumor immunity. In contrast, in the neoadjuvant setting of NSCLC patients with locally advanced disease, CD137^+^CD8^+^ T cells at low levels predicted the response to therapy (27). These cells were found dysfunctional. However, the levels of CD137^+^CD8^+^ T cells increased after chemoimmunotherapy in patients with a complete pathological response, demonstrating that the triggering of the CD137/CD137L pathway induced the proper activation of cytotoxic T cells, improving cell survival and their effector functions (27).

In addition to activating immune cells, we also evaluated the levels of immune suppression by monitoring the changes in circulating Tregs and MDSCs induced by ICIs and correlating these cells with the activated counterparts. Interestingly, immunotherapy positively affected the immune system of cancer patients, inducing a significant increase in the non-suppressive Treg subset and a decrease in the active Treg population. Due to the opposite trends of these two cellular subsets, no modulation in the total Tregs was obtained. The non-suppressive Treg appeared to correlate with Ki67^+^CD137^+^ T cells, suggesting that the activated immune cells could modulate the immunosuppressive counterpart, probably influencing the development of Tregs with no suppressive activity. Moreover, analyzing the Tregs/CD137^+^ T cell ratio, we observed that these values were lower in responding patients at baseline and during treatment, confirming the presence of a high level of circulating CD137^+^ T cells in this group of patients. Due to the immunosuppressive functions of Tregs, most authors correlated the high levels of Tregs, both circulating or infiltrating, with poor survival (34). However, data regarding Tregs in NSCLC and other solid tumors were controversial. Indeed, several authors demonstrated that increasing levels of Tregs correlated with good survival. In our previous work, we demonstrated high levels of circulating Tregs in NSCLCs improved patients’ survival. However, we observed that the analysis of the balance between Tregs and CD137^+^ T cells was more informative of the immunological status of cancer patients, and we proposed the ratio Tregs/CD137^+^ T cells as a biomarker of survival (16). Other authors demonstrated that high levels of Tregs in pre-treated NSCLC patients under immunotherapy correlated with longer overall survival (35). Similar results were obtained in HPV^+^ oropharyngeal cancer and colon cancer, in which a high frequency of Tregs was associated with a favorable response and positive prognostic factor, respectively (19, 36). The authors ascribed these discrepancies to the Treg functions in preventing/attenuating a prolonged immune response: the high levels of Tregs were considered indirect indicators of the power of the immune response activation. In addition, the different timing and types of therapy in which the analyses were carried out could impact the overall evaluation (37). On the other hand, the levels of PMN(Lox1^+^)-MDSCs significantly increased during therapy, suggesting that most probably ICIs did not affect this population or that their indirect efficacy on this population could be observed further.

In addition to the cellular subsets, all NSCLC patients were assessed for the release of soluble immune checkpoints which might contribute to impact the overall response against tumor. Among the soluble molecules analyzed, sPD1 was the only parameter that displayed a modulation after ICIs, showing an increase in the overall NSCLC population and the responding patients, even if the increase in this latter group was not statistically significant (p=0.06). In our previous study, we demonstrated that patients treated with nivolumab as the second line showed a decrease in the concentration of sPD1 after immunotherapy (24) demonstrating that the timing and the type of treatment administered to the patient were relevant for the release of this immune-soluble molecule. Another recent work carried out in a large cohort of NSCLC patients showed that sPD1 levels were significantly associated with better OS after 6 weeks of treatment in patients under anti-PD1 therapy alone (38) confirming that the increase of this molecule was correlated to a better prognosis probably blocking the ligation between PD1 and PDL1 expressed by cells. Despite these changes, the concentrations of sPD1 were similar at T0 and T1 between responders and non-responders, confirming that patients with a high ability to modulate the immune system, could better react against tumors. On the contrary, the level of sLAG3 did not change after therapy but it was significantly higher in non-responders both before and after immunotherapy suggesting its negative role as a predictor. Several data in NSCLC correlated high levels of sLAG3 to the early diagnosis and staging of NSCLC (39, 40) and we demonstrated that non-responding NSCLC patients treated with nivolumab had increasing levels of LAG3 after therapy confirming the results observed in naïve patients (26) such as in other solid tumors (41).

In this work, we have demonstrated that CD137^+^ T cell subsets, Treg/CD137^+^ T cell ratio, and sLAG3 evaluated at baseline could be considered potential predictive biomarkers of response to therapy. These results could have a relevant impact in the clinical setting where the PD-L1 expressed by the tumor tissue represents the only biomarker to select the best therapeutic choice for NSCLC patients. Moreover, these types of analyses are not invasive, easy to perform in the clinical setting, and overcome all the critical steps derived by the TPS evaluation (heterogeneity of PD-L1, the timing of analysis, location of the biopsy). However, the cut-off of these biomarkers correlated with the median values derived from the group of patients analyzed and needs to be validated. Our future studies will be focused on the enrollment of a large cohort of patients, which will confirm these data. On the contrary, monitoring the trend of selected immune populations (Ki67^+^, Ki67^+^CD8^+^, and non-suppressive Tregs) and soluble factors (sPD1 and sLAG3) throughout treatments is extremely informative on the efficacy of therapies, providing relevant data on the modulation of the immune system of each patient in real-time.

Beyond the limited number of patients, the heterogeneity of treatments represents another critical issue of this work. In our future research, we have planned to monitor the immunological changes of NSCLC patients treated with pembrolizumab alone, pembrolizumab plus chemotherapy, and nivolumab/ipilimumab plus chemotherapy separately to understand the impact of each treatment on the immune system of these patients. We believe this latter evaluation could be extremely useful for patients with TPS<50%, who received treatment only according to the tumor burden and patient clinical status.

In conclusion, despite the limited number of patients and the heterogeneity of treatments, we demonstrated that ICIs administered as first-line treatment changed the immunological balance of naïve NSCLC patients who received successful immunotherapy, driving the immune system of responding patients toward an active and proliferating profile. Although we demonstrated that several immune parameters could be used as predictive factors at baseline, these results underline the clinical relevance of continuously monitoring the immune system of cancer patients during therapy to define the efficacy of immunological treatment and provide evidence that these immune factors could be used as valuable biomarkers for assessing the therapeutic efficacy of ICIs in NSCLC patients.

## Data Availability

The original contributions presented in the study are included in the article/**Supplementary Material**. Further inquiries can be directed to the corresponding author.
