# Supplementary material for: Anti-PD1 therapies induce an early expansion of Ki67+CD8+ T cells in metastatic non-oncogene addicted NSCLC patients
Source: Front Immunol. 2024 Dec 18;15:1483182. doi: 10.3389/fimmu.2024.1483182 (PMC11688303; doi:10.3389/fimmu.2024.1483182)
Supplement: Supplementary file 1 [file Presentation1.pptx]

## Slide 1
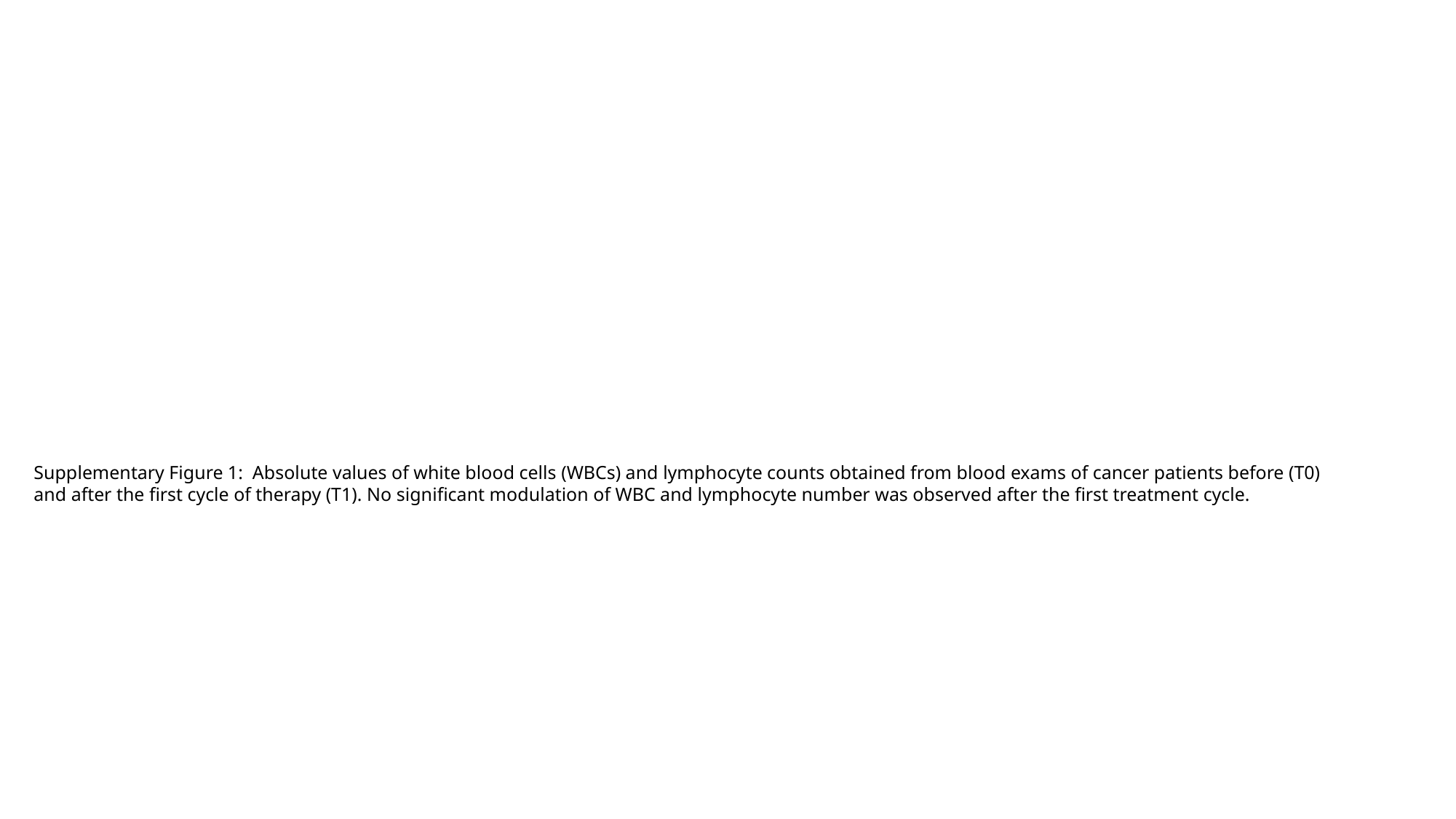

Supplementary Figure 1: Absolute values of white blood cells (WBCs) and lymphocyte counts obtained from blood exams of cancer patients before (T0)
and after the first cycle of therapy (T1). No significant modulation of WBC and lymphocyte number was observed after the first treatment cycle.

## Slide 2
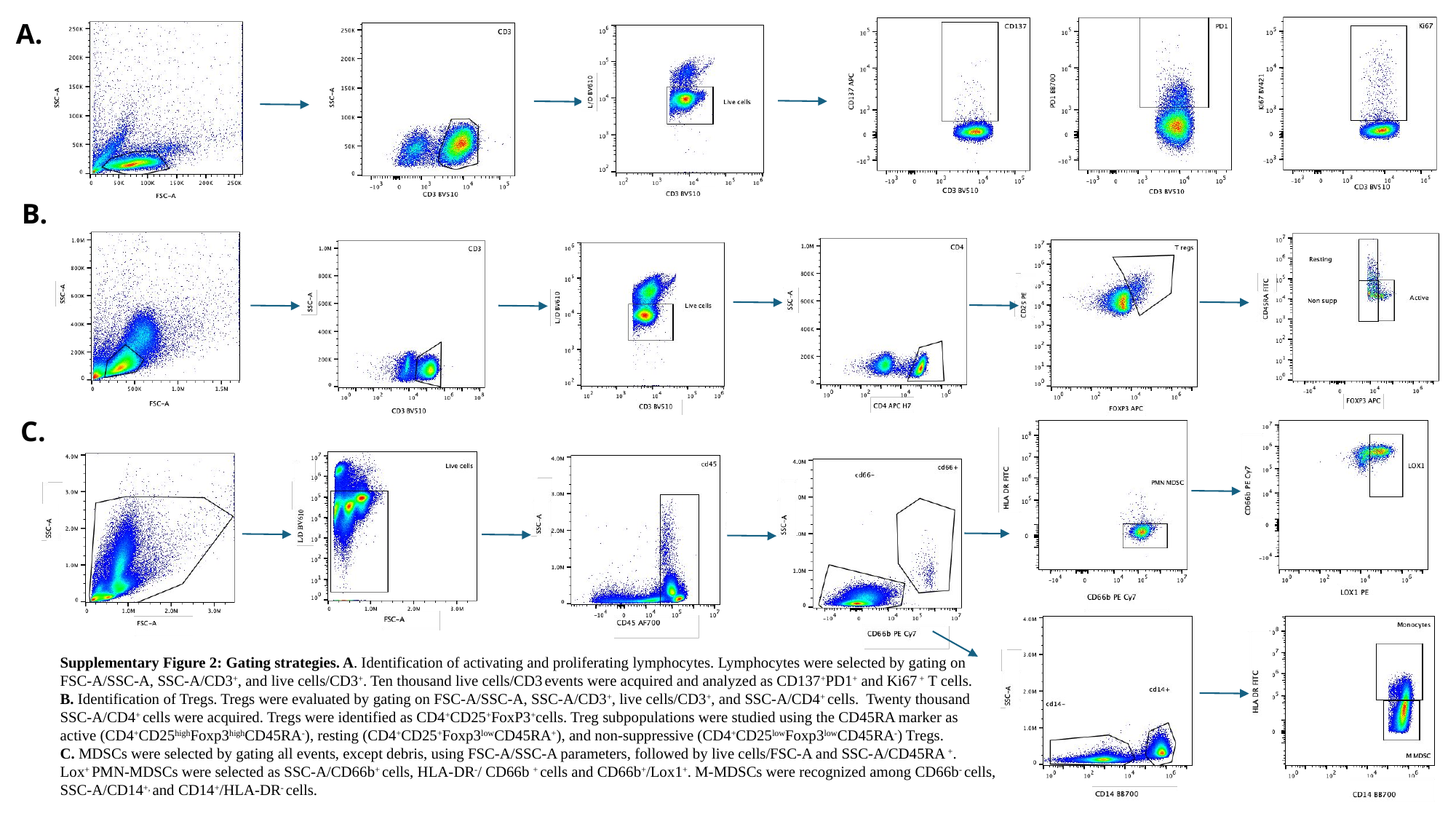

A.
B.
C.
Supplementary Figure 2: Gating strategies. A. Identification of activating and proliferating lymphocytes. Lymphocytes were selected by gating on
FSC-A/SSC-A, SSC-A/CD3+, and live cells/CD3+. Ten thousand live cells/CD3 events were acquired and analyzed as CD137+PD1+ and Ki67 + T cells.
B. Identification of Tregs. Tregs were evaluated by gating on FSC-A/SSC-A, SSC-A/CD3+, live cells/CD3+, and SSC-A/CD4+ cells. Twenty thousand
SSC-A/CD4+ cells were acquired. Tregs were identified as CD4+CD25+FoxP3+cells. Treg subpopulations were studied using the CD45RA marker as
active (CD4+CD25highFoxp3highCD45RA-), resting (CD4+CD25+Foxp3lowCD45RA+), and non-suppressive (CD4+CD25lowFoxp3lowCD45RA-) Tregs.
C. MDSCs were selected by gating all events, except debris, using FSC-A/SSC-A parameters, followed by live cells/FSC-A and SSC-A/CD45RA +.
Lox+ PMN-MDSCs were selected as SSC-A/CD66b+ cells, HLA-DR-/ CD66b + cells and CD66b+/Lox1+. M-MDSCs were recognized among CD66b- cells,
SSC-A/CD14+, and CD14+/HLA-DR- cells.
